# Glucosinolates, a natural chemical arsenal: More to tell than the myrosinase story

**DOI:** 10.3389/fmicb.2023.1130208

**Published:** 2023-04-05

**Authors:** Roula M. Abdel-Massih, Espérance Debs, Leen Othman, Jihad Attieh, Franco M. Cabrerizo

**Affiliations:** ^1^College of Medicine, Central Michigan University, Mount Pleasant, MI, United States; ^2^Department of Biology, Faculty of Arts and Sciences, University of Balamand, El-Koura, Lebanon; ^3^Faculty of Medicine and Medical Sciences, University of Balamand, El-Koura, Lebanon; ^4^Instituto Tecnológico de Chascomús, National Scientific and Technical Research Council – National University of General San Martín, Chascomús, Argentina; ^5^Escuela de Bio y Nanotecnologías, National University of General San Martín, Buenos Aires, Argentina

**Keywords:** glucosinolates, isothiocyanates, antibacterial activity, thermal degradation, chemical degradation, glucosinolate hydrolysis products, myrosinase

## Abstract

Glucosinolates are a group of thioglucosides that belong to the class of plant nitrogen-containing natural products. So far, very little biological activity has been associated with intact glucosinolates. The hydrolysis of glucosinolates has, for long, attracted attention because of the potent biological activity of the hydrolysis products. From allelopathic to antiparasitic, antimicrobial and antineoplastic effects, the activity spectrum of the degradation products of typical glucosinolates has been the subject of much research. The present review seeks to address the various means of glucosinolate degradation (thermal, enzymatic, or chemical degradation) and the ensuing products. It also aims to draw a comparative profile of the various antimicrobial effects of these degradation products to provide a further understanding of the biological function of these important compounds.

## 1. Introduction

Plants are rich in secondary metabolites such as alkaloids, polyphenols, phenolics, terpenoids and other compounds with great potential as antimicrobial agents (Othman et al., 2019). The variability in these phytochemicals is attributed to the plant’s defense mechanisms against microorganisms, insects, herbivores, or even other plants (Dangl and Jones, 2001). Humans have, for long, made extensive use of plant secondary metabolites in their various practices, such as food preparation, medication, and recreation. In recent years, the increase in microbial resistance and the slow rate of releasing new antibiotics in the market have led the scientific community to search for potential new antimicrobials hidden as secondary metabolites within plants or released from microorganisms. According to a report from the Global Research on Antimicrobial Resistance (GRAM), antimicrobial resistance is a leading cause of mortality with around 1.27 million deaths worldwide directly related to a drug-resistant infection, of which more than 70% are due to resistance to beta-lactam and fluoroquinolone antibiotics (Antimicrobial Resistance Collaborators, 2022). Moreover, many phytochemicals are regarded as safe and are favored over synthetic products as antimicrobials to help in protecting against foodborne infections (Burt, 2004; Tiwari et al., 2009; Borges et al., 2015). In this review, we will mainly focus on the antimicrobial activity of one class of nitrogen- and sulfur-containing secondary metabolites, the intact glucosinolates and their degradation products. Glucosinolates can be hydrolyzed into diverse products with different biological activities.

**SCHEME 1:**
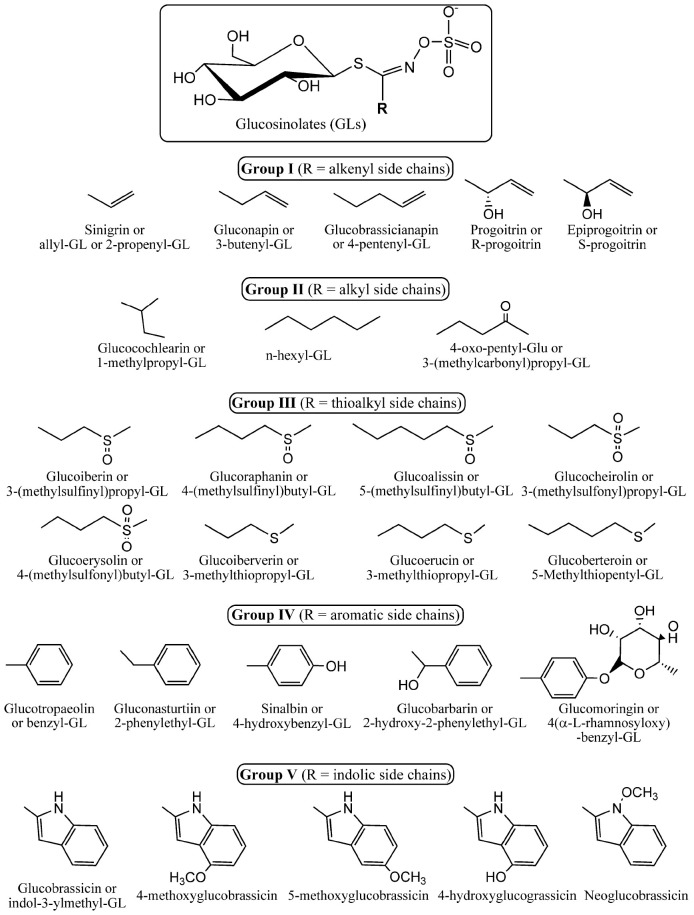
Core chemical structure of glucosinolates (top) and different side chains as the R group of the most representative glucosinolates found in nature. Glucosinolates (GLs) were classified herein as groups I to V according to the chemical nature of the side chain. They include the alkenyl, alkyl, thioalkyl, aromatic, and indolic side chains. The scheme illustrates specifically the structures of the glucosinolates that are described in the manuscript.

Glucosinolates are natural products found almost exclusively in the order Capparales, which includes around thirty families, including Brassicaceae, Caricaceae, and Capparaceae (Fahey et al., 2001). These sulfur-containing secondary metabolites occur in a variety of species such as field mustard, cauliflower, kale, garden cress, cabbage, broccoli, and similar vegetables. Glucosinolates share a common core structure consisting of a β-thioglucose linked by a sulfur atom to a (Z)-N-hydroximinosulfate ester (Halkier and Gershenzon, 2006), and a variable side chain derived from amino acids (Scheme 1).

**SCHEME 2:**
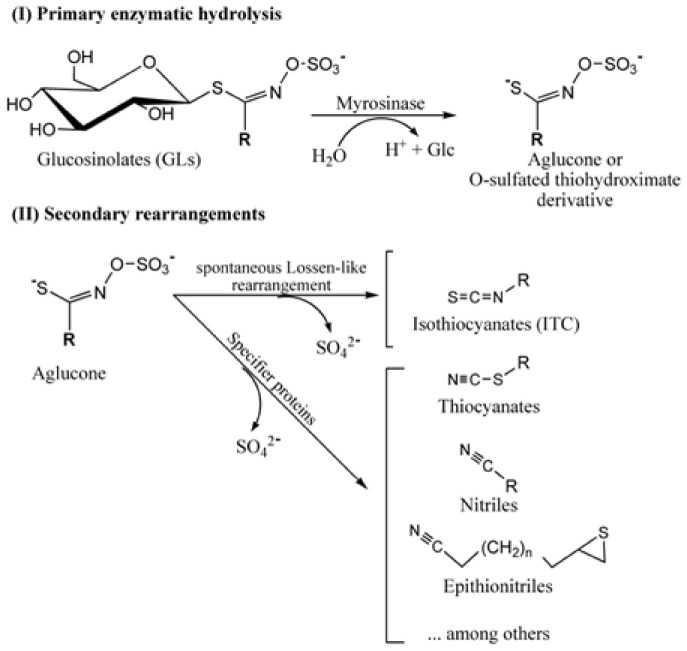
Primary hydrolytic pathway of glucosinolates catalyzed by myrosinase and secondary rearrangements yielding a diversity of products including isothiocyanates (ITCs), thiocyanates, nitriles, and epithionitriles.

Glucosinolates are particularly abundant in the Brassicaceae family and are responsible for the peculiar taste and properties of representatives of this economically important family. Only a handful of other families are known to produce these secondary metabolites. To date, more than 130 individual glucosinolate species have been identified (Blažević et al., 2020). They all have amino acid precursors, from which they are enzymatically synthesized and derive their chemical and biological properties (Nafisi et al., 2006). Glucosinolates are constitutive components of the cell, where they are synthesized early during its life, and are normally stored in the vacuole. Depending on the plant species, they can also be synthesized and sequestered in specific idioblasts, scattered in the tissue. Intact glucosinolates are known for their storage of sulfur since each molecule contains at least two atoms of this essential element. However, most of their biological activity has been attributed to glucosinolates’ hydrolysis products. Indeed, glucosinolates can be hydrolyzed into diverse products with different biological activities. Normally, they are degraded enzymatically under the effect of a thioglucosidase enzyme, commonly referred to as myrosinase (Scheme 2). The latter is endogenously separated from its substrates by compartmentalization or chemical inactivation. Upon tissue damage caused by herbivory or pathogenesis, myrosinase comes in contact with glucosinolates, and subsequently breaks them into a variety of toxic compounds, including thiocyanates, isothiocyanates, nitriles, and epithionitriles, depending on the constitution of the parent glucosinolate molecule (Scheme 2).

**SCHEME 3:**
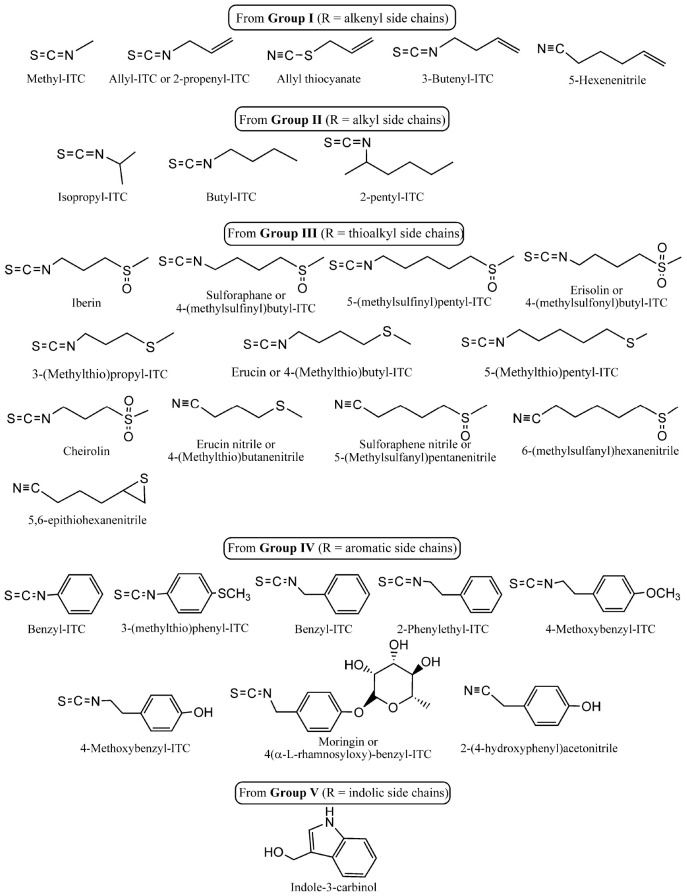
Chemical structure and classification of the main hydrolysis products discussed in the present work. Group classification is related to the parent glucosinolates shown in Scheme 1.

The potency of the hydrolysis products has been extensively researched. However, given the economic relevance of plants from the Brassicaceae family and their use in culinary and industrial practices, the degradation of constituent glucosinolates under different conditions, such as heating or chemical means, can result in alternative degradation products. These products may differ from the enzymatic products and possess potentially novel biological activities. This review focuses on the degradation of glucosinolates, rather than their biosynthesis, as the latter process has been thoroughly documented in the literature. The biosynthesis of glucosinolates begins with aliphatic, aromatic, or indole amino acids and leads to the production of a variety of glucosinolates through chain elongation (Nafisi et al., 2006; Sikorska-Zimny and Beneduce, 2021). The present review seeks to address the various means of glucosinolate degradation and the antimicrobial activity of the different hydrolysis products (Scheme 3).

## 2. Enzymatic degradation of glucosinolates

Cruciferous plants are rich in glucosinolates that are degraded into a series of products, depending on the side chains of the parent structure and the presence of different protein factors. The hydrolysis process is well documented in the literature due to its effect on the flavor, aroma, and nutritional value of the vegetables from which it is derived (Aires et al., 2009a; Clarke, 2010; Bell et al., 2018; Dejanovic et al., 2021). The plant myrosinase enzyme (E.C. 3.2.1.147), a β-thioglucosidase, cleaves the glucose group from the glucosinolate in the presence of water (Bongoni et al., 2014; Wittstock et al., 2016). Hydrolysis commonly produces isothiocyanate, which is toxic to many organisms (Burow et al., 2007). Thioglucosidases and glucosinolates are normally stored in separate compartments in plants (Rask et al., 2000) and only come in contact upon tissue damage, such as by herbivores or mechanical injury. Hydrolysis leads to the release of aglucones that rearrange spontaneously into isothiocyanates and other degradation products (Støewsand, 1995; Fahey et al., 2001; Bones and Rossiter, 2006; Aires et al., 2009a,b; Blažević et al., 2020). The formation of simple nitriles, epithionitriles, and organic thiocyanates depends on the presence of additional specifier proteins (Wittstock and Burow, 2007, 2010; Scheme 2). Nitrile specifier proteins facilitate the formation of simple nitriles (Burow et al., 2009; Kissen and Bones, 2009), epithiospecifier proteins promote the formation of epithionitriles and nitriles (Petroski and Kwolek, 1985; Lambrix et al., 2001), and thiocyanate-forming proteins have similar activity as epithiospecifier proteins but can additionally help in the formation of organic thiocyanates (Eisenschmidt-Bönn et al., 2019). Additional modifications to thiocyanates and isothiocyanates, including methylation, further potentiate these compounds and help detoxify them from the plant tissue, by volatilization (Attieh et al., 2000).

Different factors affect the hydrolysis products such as the nature of the parent glucosinolate, environmental factors, cooking preparations, proteins present, and microbiota (Verkerk et al., 2009; Barba et al., 2016). Depending on the precursor amino acids, glucosinolates can be aliphatic, aromatic, or indolic (Kliebenstein et al., 2001; Verkerk et al., 2009 thus generating different hydrolysis products. A better classification system would be to group glucosinolates according to their precursor amino acids (Ala, Glu, Ile, Leu, Met, Phe, Trp, Tyr, or Val-derived glucosinolates) or according to their degradation products (Blažević et al., 2020). The number of existing glucosinolates is variable in the literature due to the difference in identification methods and the modification in glucosinolates’ backbone or sidechains. Fahey et al. (2001) reported that more than 120 different glucosinolates have been identified. However, this number was later reconsidered due to insufficient documentation (Blažević et al., 2020). Aires and Carvalho (2017) suggested a simple method to quantify indole glucosinolate by HPLC-DAD-UV/Vis. A comprehensive critical review of the diversity of plant glucosinolates is thoroughly discussed by Blažević et al. (2020) where glucosinolate structures were fully or partially characterized by NMR. They also gave a detailed description of myrosinase and its breakdown products starting from the release of glucose (or substituted form), hydrogen ion, and a sulfated thiohydroximate that rearranges into a specific isothiocyanate (depending on the parent glucosinolate) accompanied with the release of a sulfate ion (Blažević et al., 2020). The structures and names of glucosinolates and their degradation products from mustard (*Brassica juncea*) were previously reported (Bell et al., 2018; Tian and Deng, 2020). In a more recent review, a thorough reference of the nineteen glucosinolates isolated from *Brassica rapa* L. (turnip) and their thirty-three hydrolysis products, including four epithionitriles, ten nitriles, and nineteen isothiocyanates were presented (Dejanovic et al., 2021).

Glucosinolate hydrolysis may occur due to plant or microbiota myrosinase activity (Wu et al., 2019). The endogenous plant myrosinase enzyme is inactivated by heating (Ludikhuyze et al., 1999). Inactivation may occur due to cooking at 60°C (del Carmen Martinez-Ballesta and Carvajal, 2015), stir-frying (Wu et al., 2018), microwaving (Sarvan et al., 2012), or other forms of heating or processing of cruciferous vegetables (Sikorska-Zimny and Beneduce, 2021). Hence if the plants are cooked, glucosinolates enter mostly intact into the intestines and can be degraded by gut microbiota myrosinase activity (Elfoul et al., 2001; Tian et al., 2018; Watanabe et al., 2021), since there has been presumably no human myrosinase-like activity reported. Glucosinolates are degraded into isothiocyanates, nitriles (erucin, erucin nitrile, sulforaphane, and sulforaphane nitrile), or other hydrolysis products (Lai et al., 2010; Saha et al., 2012) by colonic bacteria.

Various microorganisms, including *Aspergillus niger*, *Bacillus cereus*, *Bifidobacterium*, *Clostridium* ssp., *Geotrichum candidum*, *Lactobacillus agilis*, *Bacteroides thetaiotaomicron*, *Enterobacter cloacae*, *Enterococcus casseliflavus*, and *Escherichia coli* have been reported to metabolize glucosinolates (Oginsky et al., 1965; Ohtsuru and Hata, 1973; Tani et al., 1974; Llanos Palop et al., 1995; Elfoul et al., 2001; Krul et al., 2002; Cheng et al., 2004; Luang-In et al., 2016). Other researchers studied hydrolysis products using mixed bacterial populations isolated from human fecal samples or using a large intestinal model on glucosinolates (Combourieu et al., 2001; Krul et al., 2002). Herzallah et al. (2011) studied different factors (presence of salts) that can influence the hydrolysis of glucosinolates, such as sinigrin by pathogenic foodborne bacteria (*E. coli* O157:H7, *Staphylococcus*, *Salmonella* spp.) thus acting as antimicrobial precursors, activated by myrosinase activity, when needed. Glucosinolate microbiota degradation efficiency and hydrolysis products depend on the diet and abundance of gut bacterial strains with thioglucosidases (Angelino et al., 2015; Luang-In et al., 2016). Differences between individual fecal microbiota lead to a variation in hydrolysis product ratios (Rungapamestry et al., 2007a). Broccoli consumption increased microbiota diversity (Paturi et al., 2012) and myrosinase-like activity in the colon of rats (Liu et al., 2017) and mice (Wu et al., 2019). This increase in enzyme activity was attributed to the presence of *Clostridiaceae*, *Lachnospiraceae*, and *Porphyromonadaceae* species (Wu et al., 2019). Watanabe et al. (2021) suggested that a phosphorylation step is required for hydrolysis to occur. When comparing glucosinolate hydrolysis products by plant and microbial myrosinase, the latter resulted in significantly lower isothiocyanate concentrations, suggesting further breakdown or sequestration (Rouzaud et al., 2003). Further studies are still needed to fully understand the mechanisms for glucosinolate metabolism by gut bacteria.

## 3. Thermal degradation of glucosinolates

The glucosinolate-myrosinase system in *Brassica* plants can be thermally altered on multiple levels, due to heating or cooking. Heat may result in total or partial inactivation of myrosinase, seeping of glucosinolates or their metabolites into the cooking medium, loss of enzymatic cofactors, and thermal degradation or volatilization of metabolites (Rungapamestry et al., 2007b). The degree of change can be correlated to the method and duration of cooking, the extent of cellular disruption, and the structure and stability of the glucosinolate precursors (Rungapamestry et al., 2007b). Interestingly, myrosinase activity showed variability within and between *Brassica* species and even in different plant parts (Rungapamestry et al., 2007b). It takes around 3 min of heating at 60°C for the myrosinase extracted from broccoli to lose over 90% of its activity (Ludikhuyze et al., 1999), whereas it required 30 min at 70°C to inactivate myrosinase, extracted from red or white cabbage to the same degree (Yen and Wei, 1993). Variability was also observed in different methods of thermal treatment. Thermal treatment of *Brassica* species by steaming, stir-frying, and microwaving showed no significant decrease in the concentrations of glucosinolates in some studies. However, boiling demonstrated a loss by approximately 90% in total glucosinolate content (Barba et al., 2016).

Changes in total glucosinolate concentration after heating may occur mostly due to the leaching of glucosinolates into the cooking medium, following cell lysis. The degree to which glucosinolates are lost into the medium is affected by the size of cut pieces, the temperature of the cooking medium, and the duration of cooking (Rungapamestry et al., 2007b). Sones et al. (1984) and Rosa and Heaney (1993) reported a glucosinolate loss of 28 and >50%, respectively, after boiling cabbage in water for 10 min. Cooking green cauliflower and purple cauliflower showed a significant decrease in glucosinolate content by 68.9 and 69.2%, respectively, in comparison to their raw counterparts, whereas only a 6% decrease was observed in rutabaga. Hydrothermal processing showed a decline in total isothiocyanates and indoles content by 11 and 48.5%, respectively, in green cauliflower, 42.4 and 75.8% in purple cauliflower, and conversely, an increase of 329.4 and 142.9%, respectively, in rutabaga (Kapusta-Duch et al., 2016). This increase was attributed to the different morphology of rutabaga (root) compared to that of cauliflower (Kapusta-Duch et al., 2016).

Boiling Brussels sprouts for 5 min significantly decreased the indole glucosinolates 4-hydroxyglucobrassicin (63%) and 5-methoxyglucobrassicin (53%) in comparison to raw vegetables. The variation in loss was also attributed to the different chemical structure and thermal lability of aliphatic *versus* indole glucosinolates (Ciska et al., 2015). According to Ciska and Kozłowska (2001) the total loss of indole glucosinolates after boiling cabbage for 10–30 min was 5–10% higher than that of aliphatic glucosinolates. It has been established that indole glucosinolates are more susceptible to leaching than their aliphatic counterparts (Rungapamestry et al., 2007b; Zinoviadou and Galanakis, 2017). Pretreatment of wild rocket leaves caused a glucosinolate content reduction of 10 and 34% when treated with hot water and steaming, respectively. Both treatments caused a significant reduction in glucoraphanin, glucothiobeinin, and glucosatibin. However, steaming reduced the concentration of these glucosinolates much more than pretreatment with hot water. In contrast, hot water pretreatment significantly reduced glucobrassicin, dimeric 4-mercaptobutyl and 4-methoxyglucobrassicin (Radziejewska-Kubzdela et al., 2019). Volden et al. (2008) studied the effect of various thermal processing procedures on individual glucosinolates in red cabbage. The total aliphatic glucosinolate content was 78% (with glucoraphanin as the major component) and indole glucosinolates accounted for 19% of total glucosinolates (with 4-methoxyglucobrassacin as the major component). The reduction in glucosinolate content was highest in blanched (64%), followed by boiled (38%), then steamed vegetables (19%). The reduction in aliphatic compounds after blanching was 66%, while it was 61% in aromatic and 67% in indole glucosinolates. Boiling reduced the aliphatic, aromatic and indole glucosinolates by 40, 28, and 42%, respectively. Comparatively, steaming reduced aliphatic glucosinolates by 22% and indole glucosinolates by 25%. Most loss affected 4-methoxyglucobrassicin, glucoerucin and progoitrin as compared to other glucosinolates (Volden et al., 2008).

Slominski and Campbell (1989) evaluated the indole glucosinolate content and the thermal breakdown products in Brassica vegetables. Thermal treatment resulted in significant breakdown of indole glucosinolates into indole acetonitriles and thiocyanate ion that accounted for 30 and 50% of degraded products, respectively. In contrast, autolysis (enzymatic digestion of raw vegetables with myrosinase) resulted in almost no production of indole acetonitriles but significant production of thiocyanate ions and other byproducts (Slominski and Campbell, 1989). MacLeod and MacLeod (1970) suggested that cyanides are likely produced as a result of a thermal degradation of glucosinolates such as sinigrin. Hifnawy et al. (2013) found a decrease in the isothiocyanate ratio and an increase in cyanides after inactivation of myrosinase and thermal treatment of *Brassica oleraceae* samples.

Studies have indicated that indolic glucosinolates are generally more vulnerable to thermal degradation compared to aliphatic or aromatic glucosinolates (Fahey et al., 2001; Verkerk et al., 2009; Zhang et al., 2017). Verkerk et al. (2009) observed that glucobrassicin, an indolic glucosinolate, degraded completely after being heated at 100°C for 20 min, while aliphatic and aromatic glucosinolates exhibited greater thermal stability, especially at temperatures below 110°C. However, at higher temperatures, the differences in stability between glucosinolates became less significant and they all underwent substantial breakdown (Oerlemans et al., 2006). Additionally, aliphatic glucosinolates were generally more susceptible to thermal degradation than aromatic glucosinolates. Verkerk et al. (2009) observed that boiling broccoli sprouts for 10 min led to almost complete degradation of aliphatic glucosinolates, while aromatic glucosinolates remained relatively stable. Similarly, Yuan et al. (2009) reported that the degradation rate of aliphatic glucosinolates in radish sprouts was higher than that of aromatic glucosinolates after cooking. However, it should be noted that the extent of thermal degradation can also be influenced by factors such as temperature, pH, and cooking method.

The bioavailability of glucosinolates and their breakdown products can be affected by preparation and processing conditions. Storing *Brassica* plants at ambient temperature and in a domestic refrigerator led to the differential loss of total glucosinolate content by 11 and 27%, respectively (Barba et al., 2016). Oliviero et al. (2012) studied the effect of water content on glucosinolate thermal degradation in broccoli. Increasing the temperature resulted in higher degradation of glucosinolates, regardless of the water content. Myrosinase was inactivated (by microwaving) in these experiments prior to treatment to rule out the effect of enzymatic breakdown. It was shown that 4-methoxyglucobrassicin had the highest degradation rate among indole glucosinolates, whereas glucoraphanin and glucoiberin had the highest degradation rates among aliphatic glucosinolates. The rate of degradation increased with the sample’s water content. This was attributed to the higher activation energy values as water content increased from 34 to 82% (Oliviero et al., 2012).

**SCHEME 4:**
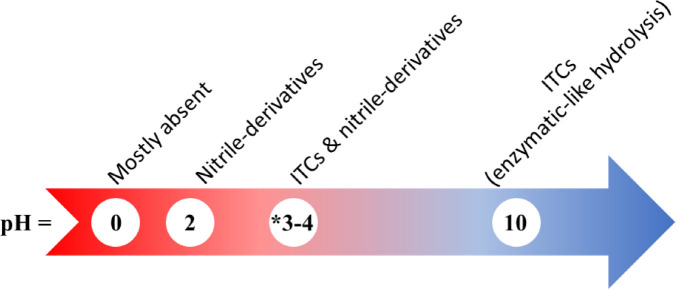
Distribution of the main glucosinolate hydrolysis products (isothiocyanates (ITCs), nitrile derivatives, or other products) as a function of pH. As reported by Blažević et al. (2014, 2015), Hanschen (2020). *The pH of a vinegar solution is estimated to be around pH 3-4.

The degradation rate of glucosinolates is also affected by the acidity of the heating media. Vinegar significantly increased the formation of isothiocyanates in cabbage salad from 0.09 to 0.21 μmol/g of raw weight, whereas lemon juice (less acidic) had a lower effect (Hanschen, 2020). It was also found that while glucosinolates were relatively stable when boiled in neutral medium, boiling in acidic medium increased glucosinolate degradation and nitrile formation (Scheme 4). Accordingly, for a diet rich in isothiocyanates it was advised to acidify raw cabbage (Hanschen, 2020).

Other factors affecting degradation of glucosinolates include storage conditions, shredding, and cooking (Verkerk et al., 2009). Song and Thornalley (2007) studied these conditions on seven major glucosinolates in Brussels sprouts, broccoli, cauliflower, and green cabbage. Broccoli had the highest glucosinolate content, with approximately 62 μmol/100 g of fresh weight, while green cabbage had the lowest content, with approximately 10 μmol/100 g. When stored at room temperature (12–22°C) for 7 days, there was no significant decrease in the glucosinolate content of *Brassica* vegetables. However, storing at 4–8°C (refrigerator) for 7 days, showed a decrease in glucosinolate content. There were only minor changes in the first 3 days of storage, then there was an 11–27% decrease in total glucosinolate content. The loss of glucoraphanin, glucoiberin, and glucoalyssin was higher than that of sinigrin, progoitrin, and gluconapin. Further, storage of *Brassica* vegetables at −85°C resulted in a loss of 10–53% of total glucosinolates. This higher loss was considered due to the freeze-thaw fractionation of cells and the accessibility of myrosinase to intracellular glucosinolates (Song and Thornalley, 2007). Dicing raw vegetables into 5 mm cubes or squares showed up to a 75% decrease in glucosinolate content. This loss increased with post-shredding time and the degree to which the raw vegetables were reduced in size (Song and Thornalley, 2007). The effects of boiling, microwave cooking, steaming, and stir-frying were also examined on total glucosinolate content. Boiling (30 min) *Brassica* vegetables resulted in the highest decrease in glucosinolate content (up to 58–77%) as compared to other cooking methods (Song and Thornalley, 2007). Blanching, which is a form of short-term heating led to an increase in the isothiocyanate fraction (Hanschen et al., 2018).

Sarvan et al. (2014) developed a mathematical model to predict the different glucosinolate contents and the expected rate constant of glucosinolate degradation in foods prepared under varying conditions and temperatures. Hennig et al. (2014) applied an untargeted metabolomics approach with a subsequent random forest regression analysis to the metabolites associated with glucosinolates thermal degradation in *Brassica oleracea*. Out of a total of 413 metabolites, fifteen were considered as the degradation byproducts of glucobrassicin, six were associated with glucoraphanin degradation, while two were associated with both glucosinolates (Hennig et al., 2014).

## 4. Other hydrolysis products (chemical degradation)

Glucosinolates are composed of a thiohydroximate-O-sulfonate group linked to a glucose and either an aralkyl, alkyl, or indolyl side chain. Chemical degradation of glucosinolates can lead to different degradation products depending on the conditions of the chemical reactions. Conditions affecting glucosinolate degradation products include temperature, pH, and the presence of catalysts. The degradation products differ in their bioavailability and thus may provide different nutritional values. For instance, an indole-3-carbinol molecule was shown to condense into a polycyclic aromatic form in an acidic environment, which would affect its absorption (Barba et al., 2016).

Raney nickel-water has been used to reduce glucosinolates to amines, at high temperatures. The resulting amino groups are attached at C-0 in the glucosinolate chain; this is different from amines that arise due to catabolism, where the amino group is attached to C-1 (Bones and Rossiter, 2006). The decomposition of glucosinolates through the use of acidic catalysts produces both a specific carboxylic acid and a hydroxylammonium ion. The latter serves as a marker for the detection of glucosinolates. In comparison, alkaline glucosinolate hydrolysis leads to the formation of several byproducts, such as allyl cyanide, ammonia and thioglucose (Bones and Rossiter, 2006). For example, the reaction of potassium methoxide with 2-propenyl glucosinolate produces thioglucose (Schneider, 1912). While the basic decomposition of 4-hydroxybenzyl glucosinolate produced thiocyanate, the degradation of indol-3-ylmethyl glucosinolate (or glucobrassicin) produced sulfate, glucose, thiocyanate, H_2_S, indol-3-ylacetic acid, indol-3-ylmethyl cyanide, in addition to several byproducts (Fenwick et al., 1981).

To test the chemical degradation of glucosinolates, defatted seeds of *Lunaria annua* L. were subjected to different pH conditions: basic [Tris buffer (pH 10)], acidic (0.1 M HCl, pH 2) and highly acidic (2 M HCl), before extracting with methylene chloride (CH_2_Cl_2_) and analyzing by GC/GC-MS. The extract obtained from alkaline treatment concurred with results of enzymatic treatment. The parent glucosinolates could be identified. The extract was rich in isothiocyanates, yet the corresponding nitriles were missing or found in trace amounts (Blažević et al., 2014). The extract at pH 2 favored nitrile formation and hence had a “mixed isothiocyanate-nitrile character”; whereas for the extract at pH 0, no glucosinolate breakdown products were recovered (Blažević et al., 2014). Similarly, for the chemical degradation of *Arabis turrita* L. seeds, the treatment at pH 10 yielded glucosinolate degradation products similar to those of enzymatic hydrolysis. It was reported that basic hydrolysis of glucosinolates can lead to the formation of alkyl amino acids and 1-β-D-thioglucose by a Neber rearrangement (Friis et al., 1977). Thus, very acidic conditions (pH < 1) would not be favorable for obtaining glucosinolate breakdown products, or as a glucosinolate identification method (Blažević et al., 2014, 2015). Scheme 4 summarizes the distribution of the glucosinolate hydrolysis products according to pH.

## 5. Antimicrobial activity of glucosinolates and their hydrolysis products

Several biological activities, such as antioxidants, antimicrobials, anti-proliferative, antiviral, and anti-inflammatory agents have been attributed to glucosinolates and their hydrolysis products (Heldt and Piechulla, 2011). Glucosinolates are secondary metabolites naturally produced in response to biotic stress in Brassicaceae plants (Miao et al., 2017). The biological activity of glucosinolates is mainly dependent on their class or origin and conditions of their hydrolysis (Griffiths et al., 1998; Aires et al., 2009a). Bones and Rossiter (2006) discussed three degradation pathways for glucosinolates (enzymatic, chemical, and thermal degradation) that can lead to hydrolysis products with different properties. Maina et al. (2020) provided a comprehensive overview of different health benefits of glucosinolates and their hydrolysis products. In this review, we focus on the antimicrobial activity of glucosinolates or glucosinolate hydrolysis products obtained after thermal degradation, enzymatic degradation (whether endogenous/autolysis, exogenous myrosinase activity or gut bacterial myrosinase activity), chemical degradation, or synthetic glucosinolate degradation products. This work was expanded to include novel synthetic active hydrolysis products since the number of naturally occurring glucosinolates and their hydrolysis products are limited (Krishna et al., 2020).

Unlike intact glucosinolates, their hydrolysis products show strong antimicrobial activity (Mithen et al., 1986; Manici et al., 1997; Tierens et al., 2001; Aires et al., 2009b). Glucosinolate hydrolysis products have strong antimicrobial activity across a wide spectrum of pathogenic microorganisms (*Bacillus subtilis*, *Campylobacter jejuni*, *Candida* sp., *E. coli*, *Helicobacter pylori*, *Listeria monocytogenes*, *Pseudomonas aeruginosa*, *Salmonella typhimurium*, *Staphylococcus aureus*, *Streptococcus lactis*, *Vibrio parahaemolyticus*, among many others). Table 1 lists some of the main glucosinolate hydrolysis products with antimicrobial activity (Drobnica et al., 1967a,b; Hashem and Saleh, 1999; Lin et al., 2000a; Fahey et al., 2001; Masuda et al., 2001; Shin et al., 2004; Troncoso et al., 2005; Cartea and Velasco, 2008; Aires et al., 2009a,b; Al-Gendy et al., 2010; Jang et al., 2010; Saavedra et al., 2010; Sofrata et al., 2011; Chen et al., 2012; Dufour et al., 2012; Mushantaf et al., 2012; Borges et al., 2015; Sotelo et al., 2015; Nowicki et al., 2016; Barbieri et al., 2017; Saladino et al., 2017; Melrose, 2019; Krishna et al., 2020). In most of these studies, the vapor phase of glucosinolate hydrolysis products was found to have stronger inhibitory activity at low concentrations compared to the liquid form of these compounds (Saladino et al., 2017). Volatile glucosinolate hydrolysis products include a variety of methylated compounds, including methylthiocyanates and methylisothiocyanates. Brassicaceae, and all glucosinolate-containing plants can produce these compounds *via* a thiol methyltransferase reaction involving S-adenosyl-L-methionine as the methyl group donor (Attieh et al., 2000).

Isothiocyanates are among the most potent glucosinolate hydrolysis products known to have strong antimicrobial activity (Delaquis and Sholberg, 1997; Mithen, 2001; Borges et al., 2015; Calmes et al., 2015; Kaiser et al., 2017). Allyl isothiocyanate, benzyl isothiocyanate, erysolin (or 4-methylsulfonylbutyl isothiocyanate), 4-methoxybenzyl isothiocyanate, moringin [or4-(α-L-rhamnosyloxy)-benzyl isothiocyanate], phenyl isothiocyanate, 2-phenylethylisothiocyanate, sulforaphane (or 4-methylsulfinylbutylisothiocyanate), and other glucosinolate hydrolysis products are among some of the main reported isothiocyanates with antimicrobial activity (Table 1).

Aromatic isothiocyanates tend to have stronger inhibitory activity as compared to aliphatic isothiocyanates (Mithen et al., 1986; Manici et al., 1997). Benzyl isothiocyanates (aromatic) exhibited stronger antimicrobial activity than aliphatic isothiocyanates when tested against Gram-positive and Gram-negative bacteria (Jang et al., 2010). Sulforaphane exhibited strong antimicrobial activity on different bacteria and fungi tested (Tierens et al., 2001).

Allyl isothiocyanates, commonly found in *Armoracia lapathifolia* (horseradish) and *Wasabia japonica* (wasabi), are derived from allyl glucosinolate (sinigrin) and have been reported to have strong antimicrobial properties (Kyung and Fleming, 1997; Lin et al., 2000a,b; Masuda et al., 2001; Brandi et al., 2006; Luciano and Holley, 2009; Dufour et al., 2012). These compounds have been shown to exhibit stronger activity against fungi than bacteria (Isshiki et al., 1992; Delaquis and Sholberg, 1997), with facultative anaerobic bacteria being the least susceptible to their effects (Park et al., 2013). Among the sulfur compounds found in cabbage or sauerkraut, allyl isothiocyanate, dimethyl trisulfide, methyl methanethiosulfonate, and methyl methanethiosulfinate exhibited inhibitory activity against growth of different species of bacteria and yeasts (Kyung and Fleming, 1997). Thio-functionalized glucosinolates (glucoiberin, glucoerucin) showed stronger activity against fungi than allyl isothiocyanates (Manici et al., 2000). Sulforaphane, a highly abundant isothiocyanate found in broccoli, is derived from glucoraphanin and has been extensively studied due to its effect against *Helicobacter pylori*, a major cause of gastritis or gastric cancer (Fahey et al., 2002; Haristoy et al., 2003, 2005; Yanaka et al., 2009; Kaiser et al., 2017; Yanaka, 2017). Aires et al. (2009b) found that sulforaphane and benzyl isothiocyanate exhibit the strongest antibacterial activity among glucosinolates or degradation products tested. Indole-3-carbinol showed stronger activity against Gram-positive bacteria (Sung and Lee, 2007), indole-3-acetonitrile had more activity against Gram-negative bacteria, whereas nitriles and amines exhibited little to no activity (Aires et al., 2009b). Romeo et al. (2018) reviewed the effect of isothiocyanates against human pathogens and found allyl isothiocyanate to be the most effective against Gram-negative bacteria (specifically *E. coli*), benzyl isothiocyanate and *Moringa oleifera* isothiocyanates (moringin and its derivatives) against *S. aureus*, and sulforaphane against *H. pylori*. Allyl isothiocyanate showed a stronger activity against Gram-negative bacteria than Gram-positive bacteria; its mode of action was similar to that of polymyxin B, creating pores on the membrane leading to leakage of metabolites (Lin et al., 2000b). Interestingly, many glucosinolate degradation products showed activity against multi-drug resistant strains. Furthermore, a recent study by Ordonez et al. (2022) revealed the remarkable anti-replicative, anti-proliferative, and anti-infective activity of sulforaphane against SARS-CoV-2 and a common cold virus.

**SCHEME 5:**
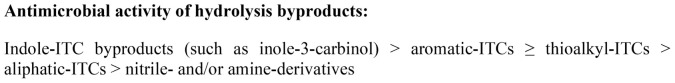
Trend of the antimicrobial activity of glucosinolates hydrolysis byproducts as a function of the side chain chemical structure. Hydrolysis byproducts include: isothiocyanates (ITCs), nitrile or amine-derivatives of glucosinolates. Indole-ITC byproducts had the strongest antimicrobial activity, followed by aromatic-ITCs and thioalkyl-ITCs, to aliphatic-ITCs, and nitrile- and/or amine-derivatives with the least activity.

Based on existing literature, the antibacterial activity of isothiocyanates varies depending on their chemical structure. Aromatic isothiocyanates, such as benzyl isothiocyanate and 2-phenylethyl isothiocyanate, have been shown to have stronger activity than aliphatic isothiocyanates (as depicted in Scheme 5). However, other factors also play a role in the antimicrobial activity of isothiocyanates, such as the length of the hydrocarbon chain, the size of the molecule, and the presence of a sulfinyl group (Wilson et al., 2013). Dias et al. (2014) demonstrated that isothiocyanates with an aromatic ring containing a shorter carbon chain are more potent than those with longer carbon chains. Therefore, benzyl isothiocyanate had stronger antibacterial activity than 2-phenylethyl isothiocyanate. This indicates that a shorter chain seems to be more efficient in killing bacteria (Dias et al., 2014). Hence, an aliphatic compound, such as 3-(methylthio)propyl isothiocyanate, may exhibit stronger activity than an aromatic compound, such as phenyl isothiocyanate and 3-(methylthio)phenyl isothiocyanate (Wilson et al., 2013). Similarly, Ko et al. (2016) found that benzyl isothiocyanate and iberin had higher activities than sulforaphane, which had a shorter carbon chain. Another important structural difference is the presence of a double bond that increase potency, where sulforaphene showed stronger activity than sulforaphane. Additionally, the presence of a thiol group (-S-) gave stronger activity than a sulfinyl group (R-SO-X). Therefore, erucin exhibited stronger activity than sulforaphane (Ko et al., 2016). Ko et al. (2016) found indolyl isothiocyanates (indol-3-carbinol) to exhibit the most potent antibacterial effect, followed by aromatic isothiocyanates (benzyl isothiocyanate and 2-phenylethyl isothiocyanate), and then aliphatic sulfur isothiocyanates (erucin, iberin, and sulforaphene) against oral pathogens. Dias et al. (2014) hypothesized that benzyl-isothiocyanates may interfere with bacterial cell wall assembly and protein synthesis.

Due to the high volatility of some glucosinolate hydrolysis products, such as allyl isothiocyanate, oil or carriers are added to increase their stability and to test their ability to prevent food spoilage. Calcium alginate beads (Kim et al., 2008), maize powder (Paes et al., 2011), silica carriers (Park and Pendleton, 2012), mustard seed powder (Dai and Lim, 2014; Bahmid et al., 2020), *Saccharina japonica* carrier (Siahaan et al., 2014), *Laminaria japonica* seaweed (El Fayoumy, 2021) were used as carriers to increase the isothiocyanate’s availability. To decrease degradation, allyl isothiocyanates can be encapsulated/complexed with α- or β-cyclodextrins prior to incorporation in the matrix, forming slow release active packaging models (Kurek et al., 2017). The main volatile degradation products obtained even from the same plant may vary depending on treatment and extraction (Blaževic et al., 2010, 2011). However, a similar wide range of antimicrobial activity was observed with glucosinolate degradation products, resulting from endogenous myrosinase activity, followed by thermal degradation or heat, then subjected to exogenous enzymatic activity (Blaževic et al., 2011). Heat treatment may have further broken down some of the isothiocyanate products and altered their activity.

Different mechanisms were proposed for the antimicrobial activity of isothiocyanates and other glucosinolate degradation products (Maina et al., 2020). Depending on their structure, they can bind to SH-groups on active sites of microbial enzymes allowing the accumulation of free radicals (Jacob and Anwar, 2008; Aires et al., 2009a; Borges et al., 2015). They can also target microbial proteins causing their aggregation or loss of function (Dufour et al., 2013). Alternatively, they can affect amphipathic properties of a membrane or decrease membrane surface charge leading to bacterial death (Sofrata et al., 2011; Borges et al., 2015), block expression of specific genes responsible for quorum sensing (Jakobsen et al., 2012; Borges et al., 2014a), induce a heat shock response, act as uncouplers of oxidative phosphorylation (Kojima and Ogawa, 1971; Delaquis and Sholberg, 1997), affect peptidoglycan cell wall synthesis (Dias et al., 2014), or inhibit respiratory enzymes (Dufour et al., 2015).

Isothiocyanates can be further oxidized by cytochrome P-450 enzymes, after ingestion, to produce more reactive compounds (Davidson and Botting, 1997). It has also been reported that isothiocyanates can be degraded to amines with lower biological activity (Song et al., 2005). Human intestinal bacteria can convert intact glucosinolates to their corresponding nitriles (Cheng et al., 2004) or amines (Combourieu et al., 2001) using myrosinase-like enzymes. Many bacteria can then convert nitriles into ammonia and carboxylic acids (Brenner, 2002; Podar et al., 2005) or further metabolize amines to aldehydes, thus decreasing antimicrobial potential (Van Ginkel et al., 2008).

Variations in enzymatic, chemical, and thermal extraction or isolation methods can influence the yield of glucosinolate degradation products. Volatiles from *Armoracia rusticana* leaves and roots were isolated using hydrodistillation and microwave green methodologies (microwave assisted-distillation, microwave hydrodiffusion and gravity) (Popović et al., 2020). The primary isothiocyanate in leaves was the same regardless of the thermal degradation method employed (allyl isothiocyanate). However, yields of glucosinolate degradation products in roots varied depending on the type of thermal degradation technique employed, leading to the production of different proportions of allyl isothiocyanate, phenylethyl isothiocyanate, and nitriles. The variations in byproducts and in proportions led to differences in antimicrobial activity of the different isolates. The microwave hydrodiffusion and gravity isolate had the strongest antimicrobial activity (Popović et al., 2020).

Since variation in glucosinolate content in cruciferous plants affects the presence of glucosinolate hydrolysis products, and thus, biological activity, it is of importance to study the source of the variability. According to Cartea and Velasco (2008), aliphatic glucosinolate synthesis is regulated by the genotype, whereas environmental conditions, such as crop management, harvest, storage, and processing seem to be more important in regulating indole glucosinolate content. In a more recent study, seeds of *Brassica oleraceae* were primed with ethanol extracts from *Punica granatum* L. peel and the impact on growth and glucosinolate metabolism was studied. Interestingly, priming could result in better production of glucosinolates and their hydrolysis products (Dawoud et al., 2022). Research into the best combination of genetic and environmental conditions to yield a functional food with bioactive aliphatic, aromatic, and/or indole isothiocyanates or glucosinolate degradation products with strong antimicrobial, anticancer, or antioxidant activity is needed.

## 6. Conclusion

Glucosinolate metabolism has been at the center of much research, owing to the wide spectrum of biological activities of the hydrolysis products of these thioglucosides. Myrosinase-mediated hydrolysis and its resulting compounds received the highest research focus, and their roles as antimicrobials, antineoplastic agents, and as antioxidants are now well established. However, other means of glucosinolate degradation, such as heat or chemical treatment, still hide significant potential, since the profile of degradation products often differs from enzyme-catalyzed hydrolysis. This review aimed at collecting the available literature on the above topics, concentrating mainly on the antimicrobial abilities of the hydrolysis products. It would be of value to explore the same abilities in products obtained from heat and chemical processing of these important metabolites.

**SCHEME 6:**
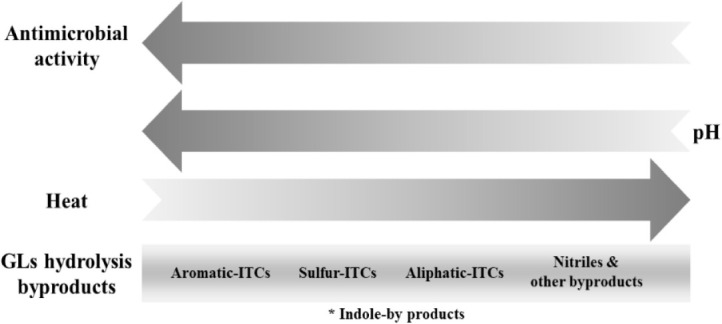
Trend of the antimicrobial activity of glucosinolates hydrolysis byproducts as a function of the pH, temperature, and chemical structure. Hydrolysis byproducts include: isothiocyanates (ITCs), nitrile or amine-derivatives of glucosinolates. *Note that the position of indole isothiocyanate byproducts in this chart may vary, as they are unstable and can spontaneously decompose to other compounds such as indole-3-carbinol, indole-acetonitrile, thiocyanate ions, and 3,3-diindolylmethane. It should be noted that the susceptibility or activity of the glucosinolates hydrolysis products may vary depending on different factors and more comparative studies are needed to have a clear scheme. Arrows point to increasing trends/concentrations.

The distribution of hydrolysis products, and hence their antimicrobial capability, is influenced by several parameters such as the nature of the parent glucosinolate, environmental factors, cooking preparations, proteins present, and microbiota. Among the hydrolysis products, isothiocyanates demonstrate the highest antimicrobial activity. However, physicochemical processes and experimental conditions like heat and pH may increase the concentration of nitrile- and amine-derivatives at the expense of isothiocyanates, thereby affecting the antimicrobial activity of hydrolysis byproducts. Therefore, the chemical nature of hydrolysis products plays a significant role in their antimicrobial activity, and the order of preference is indole-isothiocyanate byproducts (e.g., indole-3-carbinol) > aromatic-isothiocyanate > sulfur-isothiocyanate > aliphatic-isothiocyanate > nitrile- and/or amine-derivatives. The stability and concentration of these derivatives depend not only on their natural source but also on the physicochemical processes used for extracting and isolating the isothiocyanates. Scheme 6 provides an overall summary of the main observations concerning the antimicrobial activity of glucosinolate hydrolysis products, taking into account their chemical structure, temperature, and pH. However, the order of this scheme may be affected by various factors like the length of the hydrocarbon chain, the size of the molecule, and the presence of a sulfinyl group.

## Author contributions

RA-M contributed to the conception of the work, manuscript preparation, and analysis of the different manuscripts. LO contributed to the draft writing. ED and JA contributed to the concept and critically revising the work. FC contributed to the design of the work, creating the schemes, and critically revising the manuscript. All authors read and approved the final manuscript.
