# Supplementary material for: Glucosinolates, a natural chemical arsenal: More to tell than the myrosinase story
Source: Front Microbiol. 2023 Apr 5;14:1130208. doi: 10.3389/fmicb.2023.1130208 (PMC10114928; doi:10.3389/fmicb.2023.1130208)
Supplement: Supplementary file 1 [file Table_1.pdf]

Table 1. Summary of the antimicrobial activity of glucosinolates and the hydrolysis byproducts including isothiocyanates, nitriles-derivatives among others, reported in the literature. Glucosinolates were classified herein as Groups I to V according to scheme I of this manuscript or indicated if mixtures were tested.

Glucosinolate	Hydrolysis Product	Hydrolysis Method	Source	Antimicrobial Activity	Bacteria/Fungi	Reference
**[Group I]**	Allyl isothiocyanate	Commercial and/or synthetic sources (high purity)	Sigma Chemicals	Antimicrobial (studied in vapor state)	*Salmonella typhimurium*, *Escherichia coli* O157:H7, *Listeria monocytogenes*, and fungi	Delaquis and Sholberg, 1997
**[Group I]**	Allyl isothiocyanate + added to medium carrier: *Laminaria japonica* (brown seaweed)	Commercial and/or synthetic sources (high purity)	Sigma-Aldrich	Antibacterial activity; bactericidal effect	*Clostridium perfringens*, *Campylobacter jejuni*	El Fayoumy, 2021; Kim et al., 2008; Paes et al., 2011; Park and Pendleton, 2012; Dai and Lim, 2014; Siahaan et al., 2014
**[Group I]**	Allyl isothiocyanate	Commercial and/or synthetic sources (high purity)	Aldrich Chemical Co	Antibacterial; AITC effect was similar to that of polymyxin B	*Salmonella* Montevideo, *Escherichia coli* O157:H7, *Listeria monocytogenes*	Lin et al., 2000b
**[Group I]**	Allyl isothiocyanate (dissolved in medium chain triglyceride); microencapsulated allyl isothiocyanate	Commercial and/or synthetic sources (high purity)	Sigma	Antibacterial; tested on food	*Escherichia coli* O157:H7, *Salmonella enterica*, *Vibrio parahaemolyticus*, *Staphylococcus areus*, *Listeria monocytogenes*	Liu and Yang, 2010; Chacon et al., 2006
**[Group I]**	Allyl isothiocyanate (derived from the glucosinolate sinigrin)	Commercial and/or synthetic sources (high purity)	Aldrich Chemical	Antibacterial	*Escherichia coli* O157:H7	Luciano and Holley, 2009
**[Group I]**	Allyl isothiocyanate	Commercial and/or synthetic sources (high purity)	Sigma-Aldrich	Antibacterial	*Escherichia coli* (using heterotrophic plate count bacteria)	Mushantaf et al., 2012
**[Group I]**	Allyl isothiocyanate (vapor phase) with modified atmosphere	Commercial and/or synthetic sources (high purity)	Sigma-Aldrich Co.	Antibacterial	*Pseudomonas aeruginosa*	Pang et al., 2013
**[Group I]** Sinigrin (tested but not hydrolysed)	Allyl isothiocyanate	Commercial and/or synthetic sources (high purity)	Sinigrin from Sigma/ allyl isothiocyanate, allyl cyanide from Aldrich/	Antimicrobial	*Escherichia coli*, *Pseudomonas fluorescens*, *Aeromonas hydrophilia*, *Staphylococcus aureus*, *Bacillus subtilis*, *Pediococcus pentosaceous*, *Leuconostoc*	Shofran et al., 1998

			others were synthesized in lab		*mesenteroides*, *Lactobacillus brevis and* selected yeast	
**[Group I]** Glucosinolates from Japanese and Korean *Wasabia japonica*	Allyl isothiocyanate	Enzymatic (endogenous) and commercial sources (high purity)	Japanese and Korean *Wasabia japonica* (extracts from roots, stems and leaves); allyl isothiocyanate levels determined and extracts tested for antibacterial activity in addition to testing allyl isothiocyanate standard (Wako Pure Chemical Industries)	Antibacterial activity; leaf extracts showed strongest activity	*Helicobacter pylori* and food-borne pathogenic bacteria (*Escherichia coli* O157:H7, *Vibrio parahaemolyticus*, *Salmonella typhimurium*, *Staphylococcus aureus*, *Bacillus cereus*, *Streptococcus mutans*)	Shin et al., 2004
**[Group I]**	Major isothiocyanates released from horseradish root powder: allyl- and phenylethyl isothiocyanate; minor: 3-butenyl isothiocyanate	Enzymatic (endogenous). Horseradish powder mixed with water at 40°C for 2 h for maximum production of isothiocyanates	*Armoracia rusticana* (horseradish roots; Fluka Co.)	Antimicrobial activity: stronger against fungi than facultative anaerobic bacteria, stronger against Gram-positive than Gram-negative bacteria	Oral microorganisms, facultative anaerobic bacteria: *Streptococcus mutans*, *Streptococcus sobrinus*, *Lactobacillus casei*, *Staphylococcus aureus*, *Enterococcus faecalis*, *Aggregatibacter actinomycetemcomitans*; anaerobic bacteria: *Fusobacterium nucleatum*, *Prevotella nigrescens*, *Clostridium perfringens*; yeast: *Candida albicans*	Park et al., 2013
**[Group I]**	Allyl-, benzyl- (most active), 2-phenylethyl isothiocyanate	Not specified: AITC, BITC, and 2-PEITC (95%, 98% and 99% of purity, respectively) purified from cruciferous plants	Purified isothiocyanates from Cruciferous plant (not specified)	Antibacterial	Methicillin-resistant Staphylococcus aureus (MRSA)	Dias et al., 2014
**[Group I]** Sinigrin (different glucosinolates tested, antibacterial activity with only sinigrin)	Not determined	Pure purchased samples/ thermal degradation ; freeze dried broccoli powder extracted with boiling methanol (70%) for 10 min) for glucosinolate identification from Broccoli	Glucosinolates identified from *Brassica oleracea* (Broccoli); antimicrobial activity tested on pure glucosinolate standards (glucoiberin, sinigrin, glucotropaeolin glucobrassicin, phenethyl glucosinolate, and neoglucobrassicin) from ChromaDex (U.S.A.)	Antibacterial	*Bacillus cereus, E. coli, Salmonella typhimurium,* methicilin resistant *S. aureus*	Hinds et al., 2017
**[Groups I & II]**	Mustard flour (purchased, local company in Baltimore; contains allyl	Commercial and/or synthetic sources (high purity); Mustard flour	Aldrich Chemical Co	Antibacterial, bactericidal	Foodborne pathogens: rifampicin-resistant strain of *Salmonella* Montevideo, streptomycin-resistant	Lin et al., 2000a; Park et al., 2000;

	isothiocyanate); allyl and methyl isothiocyanate				strains of *Escherichia coli* O157:H7 and *Listeria monocytogenes*; *Salmonella enterica* serovar Typhimurium	Rhee et al., 2003
**[Groups I & IV]** Sinigrin (2-propenyl glucosinolate), glucotropaeolin, gluconasturtiin	Isothiocyanates: allyl-, benzyl-, 2-phenylethyl-; nitriles⁄cyanides: allyl-, benzyl-, 2-phenylethyl-, indole-3-acetonitrile; amines: allyl-, benzyl-, 2-phenylethyl-amines, and indole-3-carbinol	Commercial and/or synthetic sources (high purity)	Purchased from Sigma-Aldrich, LKT Labs; glucotropaeolin and gluconasturtiin were a gift from Prof. Dr Renato Iori (CRA-ISCI, Italy), enzymatic degradation	Antibacterial: benzyl isothiocyanate and sulforaphane exhibited the strongest activity against Gram-positive and Gram-negative bacteria	Indole-3-carbinol and Indole-3-acetonitrile had some activity against Gram-positive and Gram-negative bacteria, respectively. Glucosinolate hydrolysis products showed activity against plant pathogenic bacteria (role as phytochemicals). Glucosinolates, Nitriles, and amines tested showed no activity.	Aires et al., 2009a; Aires et al., 2009b
**[Groups I & IV]**	Allyl-, 2-phenylethyl isothiocyanate	Commercial and/or synthetic sources (high purity)	Sigma-Aldrich	Antibacterial; prevent/control biofilm formation and bacterial motility	*Escherichia coli*, *Pseudomonas aeruginosa*, *Staphylococcus aureus*, *Listeria monocytogenes*	Borges et al., 2014a; Borges et al., 2014b
**[Groups I & IV]**	Allyl-, 2-phenylethyl isothiocyanate	Commercial and/or synthetic sources (high purity)	Sigma-Aldrich	Antibacterial	*Escherichia coli*, *Pseudomonas aeruginosa*, *Staphylococcus aureus*, *Listeria monocytogenes*	Borges et al., 2015
**[Groups I & IV]**	Allyl-, benzyl-, 2-phenylethyl isothiocyanate	Commercial and/or synthetic sources (high purity)	Sigma-Aldrich	Antibacterial; synergy with some antibiotics	*Escherichia coli*, *Pseudomonas aeruginosa*, *Staphylococcus aureus*, *Listeria monocytogenes*	Saavedra et al., 2012; Abreu et al., 2013
**[Groups I & IV]** Sinigrin and gluconasturtiin (2-phenylethyl glucosinolate); horseradish root powder was treated with β-thioglucoside glucohydrolase enzyme to release isothiocyanates	Allyl- (60%), phenylethyl- (36%), 3-butenyl isothiocyanate (1.5%)	Enzymatic (exogenous)	*Armoracia rusticana* (horseradish) root	Antifungal	Pathogenic dermal fungi: *Trichophyton rubrum*, *Trichophyton mentagrophytes*, *Microsporum canis*, *Epidermophyton floccosum*	Choi et al., 2017
**[Groups I & IV]**	Allyl-, 2-phenyl isothiocyanate	Thermal degradation. Horseradish distillates [distillate contained 90% allyl isothiocyanate and 9% of 2-phenethyl isothiocyanate; trace amounts of allyl thiocyanate]; thermal degradation (20 min after boiling)	Horseradish (*Armoracia rusticana*) root	Antibacterial; horseradish distillates exerted variable bacteriostatic and bactericidal effects towards different bacterial strains on agar and cooked roast beef surfaces	*Staphylococcus aureus*, *Escherichia coli* O157:H7, *Salmonella typhimurium*, *Listeria monocytogenes, Serratia grimesii*, *Lactobacillus sake*	Ward et al., 1998
**[Groups I & IV]**	Allyl-, benzyl- (most active), 2-phenylethyl isothiocyanate	Not specified: AITC, BITC, and 2-PEITC (95%, 98% and 99% of	Purified isothiocyanates from Cruciferous plant (not specified)	Antibacterial	Methicillin-resistant *Staphylococcus aureus*	Dias et al., 2014

		purity, respectively) were purified from cruciferous plants				
**[Groups I & IV]**	Allyl-, benzyl isothiocyanate	Commercial and/or synthetic sources (high purity)	Sigma-Aldrich	Antibacterial (bactericidal rather than bacteriostatic)	*Campylobacter jejuni*	Dufour et al., 2012
**[Groups I & IV]**	Allyl-, benzyl-, phenylethyl isothiocyanate; nature-identical isothiocyanate mixture	Commercial and/or synthetic sources (high purity). The phytomedical preparation Angocin® is made of 38% (v/v) AITC, 50% BITC, and 12% PEITC to correspond to horseradish and nasturtium glucosinolates/byproducts	Sigma-Aldrich; "nature-identical isothiocyanate mixture" Angocin® purchased to mimic isothiocyanates derived from nasturtium (*Tropaeolum majoris*) and horseradish (*Armoracia rusticana*)	Antibacterial; inhibit biofilm formation; combining with meropenem increased antibacterial activity	Gram-negative bacteria such as *Pseudomonas aeruginosa*	Kaiser et al., 2017
**[Groups I, II & III]** Sinigrin, progoitrin, glucoiberin, 4-oxopentyl glucosinolate, glucocheirolin and glucoerysolin tested	Different glucosinolate hydrolysis products obtained through natural autolysis or exogenous myrosinase hydrolysis such as cheirolin (from glucocheirolin) and iberin (from glucoiberin). Allyl isothiocyanate (from sinigrin) was detected only after natural autolysis	Enzymatic (endogenous and exogenous)	*Erysimum cornithium* Boiss (Brassicaceae)	Antimicrobial: glucocheirolin from seeds showed stronger antimicrobial activity than glucosinolates from leaves or roots	*Escherichia coli*, *Bacillus subtilis*, *Staphylococcus aureus*, *Streptococcus faecalis*, *Neisseria gonorrhea*, *Pseudomonas aeruginosa*, *Candida albicans*	Al-Gendy et al., 2010
**[Groups I, II & IV]**	Methyl-, allyl-, benzyl isothiocyanates	Commercial and/or synthetic sources (high purity)	Sigma-Aldrich Chemical Co.	Antifungal (fungitoxic)	*S. sclerotiorum;* methyl, allyl and benzyl isothiocyanate were the most fungitoxic against *S. sclerotiorum*. Aromatic isothiocyanates (phenyl, benzyl and 2-phenylethyl isothiocyanates) less toxic in petri dishes (volatile form) but more toxic when dissolved in the agar than alkenyl aliphatic forms (methyl, allyl, butyl and ethyl isothiocyanates)	Kurt et al., 2011
**[Groups I, II & IV]** Sinigrin (main), glucocochlearin, glucotropaeolin, 4-methoxyglucobrassicin	Allyl isothiocyanate (from sinigrin)	Thermal and/or enzymatic vs commercial standard (high purity). Glucosinolate hydrolysis products (volatiles) obtained either by thermal (hydrodistillation) or by enzymatic degradation (treatment with myrosinase for 24 h followed by dichloromethane extraction). Comparison with main product pure allyl isothiocyanate (Sigma).	*Lepidium latifolium* L.; Sigma-Aldrich	Antimicrobial	Pathogenic and food spoilage bacteria (standard and clinical strains). Gram-positive bacteria: *Listeria monocytogenes*, *Staphylococcus aureus*; Gram-negative bacteria: *Salmonella typhimurium*, *Escherichia coli*, *Pseudomonas aeruginosa*, *Acinetobacter baumannii*. Fungus: *Candida albicans*	Blažević et al., 2019

**[Groups I, III & IV]**	Sulforaphane, phenyl-, allyl-, benzyl-, isopropyl isothiocyanate.	Not specified (start antimicrobial assay with 0.25 to 32 mM, high purity)	All plant derived except for isopropyl isothiocyanate. Isothiocyanates inhibit bacterial growth without production of Stx toxin and inhibit the stx-harboring prophages' lytic development	Antibacterial	Enterohemorrhagic *Escherichia coli* (Shiga toxin-producing *E. coli* strains)	Nowicki et al., 2014 & 2016
**[Group II]**	Aliphatic isothiocyanates and derivatives	Commercial and/or synthetic sources (high purity)	Sigma; synthetic isothiocyanate compounds	Antimicrobial	*Mycobacterium tuberculosis*	Kurepina et al., 2013
**[Groups II & IV]**	Benzyl- followed by 2-phenylethyl isothiocyanate (aromatic isothiocyanates) greater antimicrobial activity than aliphatic isothiocyanates 3-butenyl and 4-phentenyl isothiocyanate	Commercial and/or synthetic sources (high purity)	Purchased from Kasei (Japan)	Antimicrobial (stronger against Gram-positive bacteria)	Gram-positive bacteria: *Bacillus cereus*, *Bacillus subtilis*, *Listeria monocytogenes*, *Staphylococcus aureus*; Gram-negative bacteria: *Aeromonas hydrophila*, *Pseudomonas aeruginosa*, *Salmonella choleraesuis* KCCM, *Salmonella enterica*, *Serratia marcescens*, *Shigella sonnei*, *Vibrio parahaemolyticus*	Jang et al., 2010
**[Group III]** Arabidopsis extracts fractionated to give glucosinolates; glucoraphanin (or 4-methylsulfinylbutylglucosinolate)	4-methylsulfinylbutyl isothiocyanate	Thermal degradation	*Arabidopsis* plants	Antimicrobial	Wide range of bacteria: *Erwinia carotovora*, *Escherichia coli*, *Pseudomonas syringae*, *Sarcina lutea*, *Xanthomonas campestris*. Fungi: *Alternaria brassicicola*, *Botrytis cinerea*, *Fusarium culmorum*, *Fusarium oxysporum*, *Nectria hematococca*, *Neurospora crassa*, *Penicillium expansum*, *Plectosphaerella cucumerina*, *Verticillium dahliae*, *Peronospora parasitica*	Tierens et al., 2001
**[Group III]** Glucoraphanin	Sulforaphane	Enzymatic (exogenous)	Broccoli sprouts (*Brassica oleracea*)	Antibacterial	3 reference strains and 45 clinical isolates *H. pylori*; bacteriostatic [minimal inhibitory concentration (MIC) for 90% of the strains is <4 ug/ml]	Fahey et al., 2002
**[Groups III & IV]**	Benzyl isothiocyanate, sulforaphane or 4-(methylsulfinyl) butyl isothiocyanate], 2-phenylethyl isothiocyanate were most effective	Commercial and/or synthetic sources (high purity)	Commercial sources or prepared by chemical synthesis	Antibacterial	*Aeromonas* species isolated from intestinal segments of pigs	Aires et al., 2013
**[Group III from fractionated mixture]**	Extraction after hydrolysis with exogenous myrosinase: from glucoraphanin [4-(methylsulfinyl)	Enzymatic (exogenous), followed by CH_2Cl_2 extraction	*Cardaria draba* L. Desv.	Antimicrobial	Wide range of activity against Gram-positive and Gram-negative bacteria and fungi: *E. coli*, *K. pneumonia*,	Radonic et al., 2011

Glucoraphanin (4-methylsulfinylbutylglucosinolate), and smaller amounts of glucosinalbin, glucoerysolin [from extract II]	butyl isothiocyanate (69%) and 5-(methylsulfinyl) pentanenitrile] (4.5%); from glucosinalbin [2-(4-hydroxyphenyl) acetonitrile] (7%); from glucoerysolin [4-(methylsulfonyl) butyl isothiocyanate] (5.0%)				*Enterobacter sakazakii*, *P. aeruginosa*, *Cronobacter spp.*, *S. aureus*, *Rhizopus stolonifera*, etc.	
**[Group IV]** Gluconasturtiin	Phenylpentyl isothiocyanates (from horseradish autolysates and synthesized isothiocyanates)	Enzymatic (autolysis/endogenous) and synthetic (high purity) [a series of ω-phenylalkyl isothiocyanates synthesized starting from commercially available chemicals]	*Armoracia rusticana* (horseradish); synthetic isothiocyanates	Antimicrobial; phenylpentyl isothiocyanate and benzyl isothiocyanate showed strong antimicrobial activity. First report on the antimicrobial activities of phenylpropyl isothiocyanate, phenylbutyl isothiocyanate, phenylpentyl isothiocyanate; phenethyl isothiocyanate had strongest antifungal activity	Activity against Gram-positive and Gram-negative bacteria and fungi: *Staphylococcus aureus, Bacillus subtilis, B. cereus, Salmonella enterica, Proteus vulgaris, E. coli, C. albicans, Aspergillus brasiliensis*	Dekic et al., 2017
**[Group IV]**	Phenyl isothiocyanate	Commercial and/or synthetic sources (high purity)	Sigma	Antibacterial; affected adhesion, motility, and biofilm formation in bacteria; promising disinfectant	*Escherichia coli* and *Staphylococcus aureus*	Abreu et al., 2013; Abreu et al., 2014
**[Group IV]**	Sulforaphane	Commercial and/or synthetic sources (high purity)	Synthesized at University of Minnesota Cancer center	Pathogenic bacteria (role as phytochemicals)	Activity against broad spectrum of bacteria and fungi (23 out of 28 microbial species tested). *E. coli*, *Klebsiella pneumoniae*, *Salmonella typhimurium*, *Shigella sonnei*, *Enterobacter cloacae*, *Proteus vulgaris*, *Pseudomonas aeruginosa*, *Neisseria sicca*, *Pasteurella multocida*, *Staph.* sp., *Streptococcus pyogenes*, *Enterococcus faecalis*, *Bacillus* sp. Fungi: *Candida albicans*, *Crypt. neoformans*	Johansson et al., 2008
**[Group IV]** Glucosinolates from horseradish (*A. lapathifolia* Gilib.)	Phenethyl isothiocyanate (identified and isolated)	Enzymatic (endogenous)	*Armoracia lapathifolia* Gilib. (horseradish) roots	Antimicrobial	Bacteria: *Xanthomonas axonopodis* pv. Citri. Fungi: *Cytospora* spp., *Phytophthora capsici*, *Gibberella zeae*	Chen et al., 2012
**[Group IV]** Glucomoringin [4-(α-L-rhamnosyloxy) benzyl glucosinolate]	Moringin [(4-(α-L-rhamnosyloxy)-benzyl isothiocyanate)]	Enzymatic (exogenous)	*Moringa oleifera* Lam.	Antimicrobial	*Staphylococcus aureus*, *Enterococcus casseliflavus*	Galuppo et al., 2013.

**[Group IV]**<br>*Sinapis alba* L. glucosinolate	Phenethyl isothiocyanate	White mustard seeds ground to a fine powder/ Purchased (high purity)<br>Enzymatic (endogenous) + subjected to supercritical fluid extraction to obtain essential oil (thermal degradation)	*Sinapis alba* L. (white mustard) seed-derived phenethyl isothiocyanate; other isothiocyanates tested purchased from Fluka or Aldrich	Antibacterial; aromatic isothiocyanates (with phenethyl-, benzyl-, and benzoyl- groups) showed stronger activity than aliphatic isothiocyanates (with acetyl-, allyl-, butyl-, ethyl-, and methyl groups)	Antibacterial activity only against some pathogenic intestinal bacteria (*Clostridium difficile*, *E. coli*)	Kim and Lee, 2009
**[Group IV from fractionated mixture]**<br>Sinalbin or 4-hydroxybenzyl glucosinolate	4-hydroxybenzyl isothiocyanate	Enzymatic (endogenous) - essential oil	*Sinapis alba* L. (white mustard) seed	Antibacterial added to sauce with *Salmonella* inoculated particulates	*Salmonella* sp.	David et al., 2013
**[Group IV from fractionated mixture]**	Benzyl isothiocyanate, chloroform fraction of alcohol extraction and essential oil fraction of *Tropaeolum pentaphyllum* Lam. Tubers (mainly composed of benzyl isothiocyanate)	Enzymatic (endogenous)/ extraction and fractionation [tubers air dried and extracted with 70% ethanol solution followed by different solvents; essential oil was also studied	*Tropaeolum pentaphyllum* Lam. Tubers (Tropaeolaceae)	Essential oils and the chloroform fraction (of the 70% hydroalcoholic extract) showed strongest antimicrobial activity<br><br>Benzyl isothiocyanate identified by GC-MC<br>Standard tested exhibited strong antifungal activity<br><br>Chloroform fraction has additional active components	Different strains of Gram-positive and negative bacteria, *Candida* spp., and dermatophytes. Strongest activity against *E. coli* and *S. pullorum*, fungi (except some filamentous fungi), and fluconazole-resistant yeasts	da Cruz et al., 2016
**[Group IV from fractionated mixture]**<br>Glucosinolates from *Hippocratea welwitschii* roots	Aromatic isothiocyanates (benzyl isothiocyanate and 4-methoxybenzyl isothiocyanate)	Thermal degradation; essential oil was extracted using steam distillation; testing oil mix, dilutions and testing isolated compounds	Essential oil of *Hippocratea welwitschii* roots	Antibacterial	Pathogenic bacteria: *Staphylococcus aureus*, *Streptococcus pyogenes*, *Klebsiella pneumoniae*, *Bacillus subtilis*, *Escherichia coli*, *Shigella dysenteriae*, and *Pseudomonas aeruginosa*	Iwu et al., 1991
**[Group IV from fractionated mixture]**	Benzyl isothiocyanates	Thermal degradation; fractionation and testing of separate fractions and combination to verify if antibacterial effect is due to benzyl isothiocyanate; comparing with activity of commercial benzyl isothiocyanate	*Salvadora persica* (Miswak sticks) root extracts; commercial benzyl isothiocyanate (Lancaster Synthesis Inc.)	Antibacterial	Gram-negative periodontal pathogens: *Aggregatibacter actinomycetemcomitans*, *Porphyromonas gingivalis*; bactericidal effect; mechanism of action suggested is by causing protrusions in bacterial membranes	Sofrata et al., 2011
**[Group IV vs mixture and novel isothiocyanates synthetic derivatives]**	Benzyl isothiocyanate, β-phenylethyl isothiocyanate, and other isothiocyanates	Commercial and/or synthetic source (high purity). 11 natural isothiocyanates (obtained from the Royal Veterinary and Agricultura College in	11 natural isothiocyanates and 27 synthetic analogues; 57 substituted derivatives of phenylisothiocyanate	Antifungal; negative effect of ionized groups on the aromatic moiety	*Aspergillus niger*, *Penicillium cyclopium*, and *Rhizopus oryzae*	Drobnica et al., 1967a; Drobnica et al., 1967b

		Copenhagen/ details not specified/purified)/ synthesized new compounds				
**[Group V]**	Indole-3-carbinol	Commercial and/or synthetic sources (high purity)	Sigma Chemical Co., St. Louis, U.S.A.	Antifungal/antibacterial	*Staphylococcus aureus, Staphylococcus epidermidis, Escherichia coli, Saccharomyces cerevisiae, Trichosporon beigelii, Enterococcus faecium, Escherichia coli O157, Pseudomonas aeruginosa,* methicillin-resistant *Staphylococcus aureus, vancomycin-sensitive Enterococci,* multi-resistant *Escherichia coli,* multi-resistant *Pseudomonas aeruginosa,* and *Candida albicans*	Sung and Lee, 2007
**[Mixed fractions of glucosinolates and/or hydrolysis products]** Sinigrin (2-propenyl glucosinolate; allyl glucosinolate) in *Sinapis nigra*; sinalbin (4-hydroxybenzyl glucosinolate) in *Sinapis alba*	Not determined	Enzymatic (endogenous). Different solvents and methods tested	*Sinapis nigra* L. (black mustard), *Sinapis alba* L. (white or yellow mustard); ultrasound-assisted extraction of ground seeds with ethanol:water (8:2) gave highest glucosinolate yield	Antimicrobial	Gram positive: *Staphylococcus aureus*, *Streptococcus pyogenes*, *Bacillus cereus*; Gram negative: *Escherichia coli*, *Pseudomonas aeruginosa*. Fungus: *Candida albicans*	Boscaro et al., 2018
**[Mixed fractions of glucosinolates and/or hydrolysis products]** Glucoberteroin	6-(methylsulfanyl) hexanenitrile, hex-5-enenitrile, pent-4-enyl isothiocyanate, and Maillard reaction products (3,4,5-trimethyl pyrazole) may all contribute to activity	Thermal degradation/hydrodistillation-volatiles fractionated into water-soluble, water-insoluble, and high volatile fractions	*Degenia velebitica* (endangered species)	Antimicrobial; the fraction of water-soluble compounds had the strongest antimicrobial activity and the largest mass ratio among volatiles tested	*Bacillus subtilis*, *Staphylococcus aureus*, *Escherichia coli*, *Pseudomonas aeruginosa*, and *Candida albicans*	Mastelic et al., 2010
**[Mixture of glucosinolates and/or hydrolysis products]**	Nitriles, isothiocyanates, thiocyanates			Antimicrobial	Activity against broad spectrum of bacteria and fungi	Saladino et al., 2017
**[Mixture of glucosinolates and/or hydrolysis products]** Gluconapin, glucoalyssin (5-methylsulfinylpentyl glucosinolate), glucobrassicanapin (4-pentenyl glucosinolate); other glucosinolates identified in lower	The main isothiocyanates: 3-butenyl isothiocyanate (from gluconapin), 4-pentyl isothiocyanate (from glucobrassicanapin), and 5-(methylsulfinyl) pentyl isothiocyante (from glucoalyssin)	Autolysis (endogenous myrosinase) followed by hydrodistillation/heating for one batch; (heat-inactivation of endogenous enzyme) treating with exogenous myrosinase enzyme followed by CH_2Cl_2 extraction for another batch)	*Aurinia leucadea* (Guss.) C. Koch	Antimicrobial; autolysis + thermal degradation and exogenous myrosinase digestion of glucosinolates gave similar products and antimicrobial activity (although some variation, major degradation products were the same)	Wide range of food spoilage and human pathogenic microbial strains: *Bacillus cereus*, *C. albicans*, *Penicillium spp.*, *R. stolonifera*, *P. aeruginosa*	Blaževic et al., 2011

amounts: glucoraphanin, glucocochlearin, glucotropaeolin, glucoerucin, glucoberteroin						
**[Mixture of glucosinolates and/or hydrolysis products]**	Mixture of isothiocyanates (allyl-, benzyl-, phenylethyl- isothiocyanates) similar to the ones found in herbal preparation from nasturtium (*Tropaeoli majoris* herba) and horseradish (*Armoraciae rusticanae* radix) licensed native preparation; commercial compounds	Commercial and/or synthetic sources (high purity)	Angocin ® Anti-Infekt, Fluka/Sigma-Aldrich	Antibacterial	Clinical important pathogens. Bacteria: *Haemophilus influenzae*, *Moraxella catarrhalis*, *Serratia marcescens*, *Proteus vulgaris*, *S. aureus*, *S. pyogenes*, *Streptococcus pneumoniae*, *Klebsiella pneumoniae*, *E. coli*, *P. aeruginosa*. Fungus: *Candida* spp.	Conrad et al., 2013
**[Mixture of glucosinolates and/or hydrolysis products]**	Sulfur compounds (found in cabbage): allyl isothiocyanate, dimethyl trisulfide, methyl methanethiosulfinate, methyl methanethiosulfonate; sinigrin, S-methyl-L-cysteine sulfoxide, dimethyl disulfide did not show much antimicrobial activity; dimethyl sulfide showed limited activity	Commercial and/or synthetic sources (high purity)	Purchased from Sigma Chemical Company (St. Louis, MO) or Aldrich Chemical Company (Milwaukee, WI)	Antifungal/antibacterial	15 species of bacteria and 4 species of yeasts. Bacteria: *Pediococcus pentosaceus*, *Lactobacillus brevis*, *Listeria monocytogenes*, *Staphylococcus aureus*, *Escherichia coli*, *Enterobacter aerogenes*, *Bacillus subtilis*, *Salmonella typhimurium*. Yeasts: *Saccharomyces cerevisiae*, *Torulopsis etchellsii*, *Hansenula mrakii*, *Pichia membranefaciens*	Kyung and Fleming, 1997
**[Mixture of glucosinolates and/or hydrolysis products]** 17 glucosinolates (2-propenyl, 3-methylsulphinylpropyl, 4-methylsulphinylbutyl, 2-hydroxy-3-butenyl, 3-butenyl, 4-pentenyl, 4-methylthiobutyl, 4-hydroxybenzyl, 2-phenylethyl, indole-3-ylmethyl), glucosinolate hydrolysis products and methanolic extracts from glucosinolate-enriched *Brassica* crops	Glucosinolate hydrolysis products: allyl-, benzyl-, 3-butenyl-, 4-pentenyl-, phenetyl isothiocyantes, sulforafane, indole-3-carbinol	Commercial and/or synthetic sources (high purity); methanolic extracts from *Brassica* crops to obtain glucosinolates	Phytoplan Diehm and Neuberger GmbH, Sigma Aldrich, TCI Europe N. V.	Antimicrobial Activity	Tested against soil pathogens. Bacteria: *Xanthomonas campestris* pv. Campestris, *Pseudomonas syringae* pv. Maculicola. Fungi: *Alternaria brassicae*, *Sclerotinia scletoriorum*	Sotelo et al., 2015
**[Mixture of glucosinolates and/or hydrolysis products]**	Allyl isothiocyanates (endogenous myrosinase activity on *Brassica juncea* seeds)	Enzymatic (endogenous)	*Brassica juncea* (Brown Mustard)	Antimicrobial activity; allyl isothiocyanate showed potential as a vapor for food preservation	Different bacteria such as *Bacillus* spp., *Staphylococcus* spp., *Salmonella* spp., *E. coli*, *Vibrio parahaemolyticus*, and	Isshiki et al., 1992

Glucosinolates from brown mustard (*Brassica juncea*) seeds					*Pseudomonas aeruginosa*. In addition to different yeast and molds	
**[Mixture of glucosinolates and/or hydrolysis products]** Sinigrin (allyl) from *Brassica juncea* cv. Vitasso seeds; gluconapin (3-butenyl) *Brassica rapa* cv. Silla; glucotropaeolin (benzyl) *Lepidium sativum*; sinalbin (p-hydroxybenzyl) *Sinapis alba* cv. Maxi; epiprogoitrin [(2S)-2-hydroxy-3-butenyl] *Crambe abyssinica* cv. Belenzian; glucobarbarin (2-hydroxy-2-phenylethyl) *Barbarea vulgaris*; glucoerucin (4-methylthiobutyl) *Eruca sativa*; glucocheirolin (3-methylsulfonylpropyl) *Cheirantus annuus*; glucoraphenin (4-methylsulfinyl-3-butenyl) *Raphanus sativus* cv. Pegletta; glucoiberin (3-methylsulfinylpropyl) *Iberis amara*, rapeseed glucosinolate mixture. All the glucosinolates isolated from cruciferous seeds and have a purity range of 75-91% except the latter.	11 glucosinolates (isolated from some cruciferous seeds) and their myrosinase hydrolysis products	Enzymatic (exogenous)	Isolated from some cruciferous seeds	Hydrolysis products of glucosinolates (glucoiberin, glucoerucin, glucocheirolin, and glucotropaeolin; sinigrin and epiprogoitrin) had fungitoxic activity	Tested against *Fusarium culmorum* and plant pathogens *Rhizoctonia solani*, *Sclerotinia sclerotiorum*, *Diaporthe phaseolorum*, and *Pythium irregulare*	Manici et al., 1997
**[Mixture of glucosinolates and/or hydrolysis products]**	Not identified	Enzymatic (exogenous)	*Tropaeolum tuberosum*	Antifungal	*Phytophthora infestans, Fusarium oxysporum, Rhizoctonia solani*	Martin and Higuera, 2016

p-methoxybenzyl glucosinolate (main); p-hydroxybenzyl glucosinolate (not confirmed)						
**[Mixture of glucosinolates and/or hydrolysis products]** Different glucosinolates tested from *Brassica* species: sinigrin from mustard seed (*Brassica juncea*), gluconapin from turnip rapeseed (*B. campestris* cv. Sarson), progoitrin from swede seed (*B. napu* s subsp. napobrassica cv. Laurentian), glucobrassicin from young leaves of Brussels sprouts (*B. oleracea* var. gemmifera), 1-methoxyglucobrassicin from cabbage seeds (*B. oleracea* var. capitata)	2-propenyl isothiocyanate (derived from sinigrin) had the most antifungal activity; glucosinolates not identified (sinigrin + myrosinase had best activity)	Enzymatic (exogenous)	From seeds of Brassica plants: mustard seed (*Brassica juncea*), turnip rapeseed (*B. campestris*), swede seed (*B. napu*), leaves of Brussels sprouts (*B. oleracea*), cabbage seeds (*B. oleracea*)	Antifungal	*Leptosphaeria maculans*	Mithen et al., 1986
**[Mixture of glucosinolates and/or hydrolysis products]** Glucosinolates from cabbage treated with thioglucosidase (ICN Biomedical Co.) to release isothiocyanates	Allyl-, benzyl-, 2-phenylethyl- and phenyl isothiocyanates	Enzymatic (exogenous)	Cabbage	Antifungal	*Alternaria alternata* (fungus)	Troncoso et al., 2005
**[Mixture of glucosinolates and/or hydrolysis products]** *Brassica* species: sinigrin (allyl glucosinolate) as the major component (minor quantities of other glucosinolic acid derivatives). *S. alba*:	Myrosinase hydrolysis products (Chromadex, Sigma) + purchased glucosinolate hydrolysis products were tested; allyl isothiocyanate from *Brassica* species had the strongest activity	Enzymatic (exogenous). thioglucosidase enzyme from *Sinapis alba* (white mustard) seed purchased from Sigma-Aldrich added to natural extracts and to commercial glucosinolates (high purity)	*Seeds of Brassica nigra*, *B. juncea* cv. Scala, *B. carinata* cv. Eleven, *Sinapis alba* cv. Ludique	Antifungal	*Phytophthora cinnamoni* Rands, *Pythium spiculum* B. Paul	Arroyo Cordero et al., 2019

sinalbin (p-hydroxybenzyl glucosinolate or 4-hydroxybenzyl glucosinolate)						
**[Mixture of glucosinolates and/or hydrolysis products]** Glucoiberverin (3-methythiopropyl glucosinolate) (in seeds and leaves); glucoiberin (3-methylsulfinylpropyl glucosinolate) and glucoerucin (seeds)	In seeds: glucoiberverin (detected via its hydrolysis products: 3-(methylthio) propyl isothiocyanate (iberverin) and 4-(methylthio) butanenitrile (iberverin nitrile); glucoiberin (its hydrolysis product 3-(methylsulphiny) propyl isothiocyanate (iberin); glucoerucin (its hydrolysis product 4-(methylthio) butyl isothiocyanate (erucin)<br><br>In leaves: glucoiberverin (detected by its hydrolysis products, nitrile and isothiocyanate)	Enzymatic (exogeneous)	*Lobularia libyca* (Viv.) C.F.W. Meissn.	Antimicrobial	Strongest activity was against Gram positive: *Staphylococcus aureus*; Gram negative: *Pseudomonas aeruginosa*. Fungus: *Candida albicans*	Al-Gendy et al., 2016
**[Mixture of glucosinolates and/or hydrolysis products]** Twelve glucosinolates identified: sinigrin (2-propenyl glucosinolate), gluconapin (3-butenyl glucosinolate), progoitrin (2-hydroxybut-3-enyl glucosinolate), glucoerucin (4-methylthiobutyl glucosinolate), glucoiberin (3-methylsulfinylpropyl glucosinolate), glucoraphanin (4-methylsulfinylbutyl glucosinolate), glucotropaeolin (benzyl glucosinolate), glucobrassicin (indol-3-ylmethyl glucosinolate), neo-glucobrassicin (1-methoxyindol-3-ylmethyl glucosinolate), 4-methoxyglucobrassicin (4-	Extracts containing glucosinolates, isothiocyanates (allyl isothiocyanate, butane-1-isothiocyanate, etc.), nitriles, other hydrolysis products, and other active compounds (flavonoids, phenol compounds, phytoalexines) were tested as mixture	Thermal degradation. Hydrodistillation involves three main physicochemical processes: hydrodiffusion, hydrolysis, and decomposition by heat. Thermal degradation (frozen samples were heated for 15 min at 70°C)	*Brassica oleracea* L. var. Botrytis L. (Egyptian cauliflower)	Antimicrobial	Activity against broad range of Gram-positive and Gram-negative bacteria and fungi; strongest inhibitory activity against *Escherichia coli*, *Klebsiella pneumoniae*	Hifnawy et al., 2013

methoxyindol-3-ylmethyl glucosinolate), glucoiberverin (3-methylthiopropyl glucosinolate), n-hexyl glucosinolate						
**[Mixture of glucosinolates and/or hydrolysis products]**	Sulforaphane or 4-(methylsulfinyl) butyl isothiocyanate (sulforaphane) and 11 other isothiocyanates	Not specified in Harristoy et al., 2003 & 2005; enzymatic (endogenous) in Yanaka et al., 2009	Sulforaphane provided by J.W. Fahey (Haristoy et al., 2003 & 2005); Glucoraphanin-rich 3-d-old broccoli sprouts (homogenized and checked for > 60% conversion of glucoraphanin to sulforaphane by endogenous myrosinase enzyme) (Yanaka et al., 2009).	Antibacterial	25 strains of *Helicobacter pylori;* agar dilution assay + in vivo assays (mice/humans); humans were instructed to consume 70 g/d 3-d-old germinated broccoli sprouts for 8 wk (validated for glucoraphanin content)	Haristoy et al., 2003 & 2005; Yanaka et al., 2009
**[Mixture of glucosinolates and/or hydrolysis products]** Glucosinolates from horseradish	Allyl isothiocyanate, phenethyl isothiocyanate and trace amounts of allyl thiocyanate and 1-butane isothiocyanate	Thermal degradation. *Amoracia lapathifolia* root distillates	*Amoracia lapathifolia* Gilib. (horseradish)	Antimicrobial	*Staphylococcus aureus*, *Escherichia coli* O157:H7; *Salmonella typhimurium*; *Listeria monocytogenes*; *Serratia grimesii*; *Lactobacillus sake*; *Pseudomonas* spp. and Enterobacteriaceae	Ward et al., 1998; Delaquis et al., 1999
**[Mixture of glucosinolates and/or hydrolysis products]** Glucoalyssin, glucobrassicanapin, glucoberteroin	The most abundant compounds in the hydrodistillates: 6-(methylthio) hexane nitrile and 5-(methylthio) pentyl isothiocyanate (degradation products from glucoberteroin); 5-hexenenitrile and 4-pentenyl isothiocyanate (degradation products from glucobrassicanapin)<br><br>5,6-epithiohexanenitrile (from glucobrassicanapin) was only detected among hydrodistillates of fresh plant material without autolysis.<br><br>The most abundant products due to hydrolysis by exogenous myrosinase: 6-(methylsulfinyl) hexanenitrile and	Thermal degradation. Volatiles obtained from a) dry plants by hydrodistillation (thermal degradation) b) fresh plants by hydrodistillation c) from fresh plants after autolysis (endogenous enzymatic degradation) followed by hydrodistillation or d) fresh plant material – exogenous enzymatic degradation – dichloromethane extraction	*Aurinia sinuate*	Antimicrobial	Gram-positive bacteria: *Staphylococcus aureus*, *Bacillus cereus*, *Clostridium perfringens*, *Enterococcus faecalis*, *Micrococcus luteus*; Gram-negative (ampicillin-resistant) bacterial strains: *Aeromonas hydrophila*, *Chryseobacterium indologenes*, *Enterobacter sakazakii*, *Enterobacter cloacae*, *Escherichia coli*, *Klebsiella pneumoniae*, *Pseudomonas aeruginosa*, *Pseudomonas luteola*, *Vibrio vulnificus*. Fungi: *Candida albicans*, *Aspergillus niger*, *Penicillium* spp.	Blaževic et al., 2010

	5-(methylsulfinyl) pentyl isothiocyanate (alyssin) (from glucoalyssin)					
**[Mixture of glucosinolates and/or hydrolysis products]** Glucosinolates from horseradish root (*A. rusticana*)	Allyl isothiocyanate (60%), phenylethyl (36%) and a minor derivative 3-butyl isothiocyanate (1.5%) as mixture tested in vapor and liquid phase	Thermal degradation. Steam distillation	*Armoracia rusticana* (horseradish) root	Antibacterial	*Bacillus* sp., *Staphylococcus* sp., *Streptococcus* sp., and *Enterobacter* sp. isolated from jeotgal (a Korean salted and fermented seafood)	Kim et al., 2015
**[Mixture of glucosinolates and/or hydrolysis products]** Glucoerucin [from extract I]	Extraction by hydrodistillation without exogenous myrosinase treatment: 28% 4-(methylsulfanyl) butyl isothiocyanates and 14% 5-(methylsulfanyl) pentanenitrile are the main volatiles originating from glucoerucin degradation with S and N	Thermal degradation: hydrodistillation	*Cardaria draba* L. Desv.	Antimicrobial	Wide range of activity against Gram-positive and Gram-negative bacteria and fungi (*E. coli*, *K. pneumonia*, *Enterobacter sakazakii*, *P. aeruginosa*, *Cronobacter spp.*, *S. aureus*, *Rhizopus stolonifer*)	Radonic et al., 2011
**[Mixture of glucosinolates and/or hydrolysis products]** Glucosinolates from horseradish; in roots: gluconasturtiin (64.9%), sinigrin (33.91%), glucobrassicin (1.19%); in leaves: sinigrin (99.99%) with trace amounts of gluconapin and glucobrassicanapin	Allyl isothiocyanate, 2-phenylethyl isothiocyanate, nitriles (3-phenylpropanenitrile)<br><br>Similar yields in leaves but not in roots after thermal treatments	Thermal degradation: microwave hydrodiffusion and gravity (MHG); microwave-assisted distillation (green extraction and distillation techniques); microwave hydrodiffusion and gravity technique gave best activity in roots	*Armoracia lapathifolia* Gilib. (horseradish) leaves and roots; Sigma	Antibacterial	Multidrug-resistant ESKAPE strains (*Enterococcus faecium*, *Staphylococcus aureus*, *Klebsiella pneumoniae*, *Acinetobacter baumannii*, *Pseudomonas aeruginosa*, and *Enterobacter* species)	Popovic et al., 2020
**Nitrile, amines and other hydrolysis byproducts**				Glucosinolates, nitriles, and amines tested did not show antibacterial activity. Glucosinolate hydrolysis products showed activity against plants		Aires et al., 2009b; Saladino et al., 2017

REFERENCES :

Abreu, A. C., Borges, A., Simões, L. C., Saavedra, M. J., Simões, M. 2013. Antibacterial activity of phenyl isothiocyanate on *Escherichia coli* and *Staphylococcus aureus*. Med Chem. 9(5):756-61.

Abreu, A. C., Borges, A., Mergulhão, F., Manuel Simões. 2014. Use of phenyl isothiocyanate for biofilm prevention and control. International Biodeterioration & Biodegradation 86 (A): 34-41.

Aires, A., Dias, C., Rosa, E., Saavedra, M. 2013. Antimicrobial susceptibility of *Aeromonas* Spp. isolated from pig ileum segments to natural isothiocyanates. Med. Chem. 9:861-866.

Blažević, I., Đulović, A. Maravić, V. Čikeš Čulić, S. Montaut, P. Rollin. 2019. Antimicrobial and cytotoxic activities of *Lepidium latifolium* L. hydrodistillate, extract and its major sulfur volatile allyl isothiocyanate. Chemistry & Biodiversity 16(4):1612-1872.

Boscaro, V., Boffa, L., Binello, A., Amisano, G., Fornasero, S., Cravotto, G., Gallicchio, M. 2018. Antiproliferative, proapoptotic, antioxidant and antimicrobial effects of *Sinapis nigra* L. and *Sinapis alba* L. Extracts. Molecules 23, 3004.

Chacon, P.A., Muthukumarasamy, P., Holley, R.A. 2006. Elimination of *Escherichia coli* O157:H7 from fermented dry sausages at an organoleptically acceptable level of microencapsulated Allyl isothiocyanate. Appl Environ Microbiol 72:3096-3102.

Choi, K.D., Kim, H.Y., Shin, I.S. 2017. Antifungal activity of isothiocyanates extracted from horseradish (*Armoracia rusticana*) root against pathogenic dermal fungi. Food Sci. Biotechnol. 26:847-852.

da Cruz, R.C., Denardi, L.B., Mossmann, N.J., Piana, M., Alves, S.H., De Campos, M.M.A. 2016. Antimicrobial Activity and Chromatographic Analysis of Extracts from *Tropaeolum pentaphyllum* Lam. Tubers. Molecules 21, 566.

David, J.R.D., Ekanayake, A., Singh. I., Farina, B., Meyer, M. 2013. Effect of white mustard essential oil on inoculated *Salmonella* sp. in a sauce with particulates. J Food Prot 76:580–587.

Dekic, M.S., Radulovic, N.S., Stojanovic, N.M., Randjelovic, P.J., Stojanovic-Radic, Z.Z., Najman, S., Stojanovic, S. 2017. Spasmolytic, antimicrobial and cytotoxic activities of 5-phenylpentyl isothiocyanate, a new glucosinolate autolysis product from horseradish (*Armoracia rusticana* P. Gaertn., B. Mey. & Scherb., Brassicaceae). Food Chem. 232:329-339.

Galuppo, M., De Nicola, G.R., Iori, R., Dell'Utri, P., Bramanti, P., Mazzon, E. 2013. Antibacterial activity of glucomoringin bioactivated with myrosinase against two important pathogens affecting the health of long-term patients in hospitals. Molecules 18:14340-14348.

Iwu, M. M., N. C. Unaeze, C. O. Okunji, D. G. Corley, D. R. Sanson, M. S. Tempesta. 1991. Antibacterial aromatic isothiocyanates from the essential oil of *Hippocratea welwitschii* roots. Int. J. Pharmacogn. 29:154-158.

Johansson, N.L., Pavia, C.S., Chiao, J.W. 2008. Growth inhibition of a spectrum of bacterial and fungal pathogens by sulforaphane, an isothiocyanate product found in broccoli and other cruciferous vegetables. Planta Med 74:747-750.

Kim, HY., Gornsawun, G., Shin, I.S. 2015. Antibacterial activities of isothiocyanates (ITCs) extracted from horseradish (*Armoracia rusticana*) root in liquid and vapor phases against 5 dominant bacteria isolated from low-salt *Jeotgal*, a Korean salted and fermented seafood. Food Sci Biotechnol 24:1405-1412.

Kim, M.G. & Lee, H.S. 2009. Growth-inhibiting activities of phenethyl isothiocyanate and its derivatives and against intestinal bacteria. J. Food. Sci. 74:467-471.

Kurepina, N., Kreiswirth, B.N., Mustaev, A. 2013. Growth-inhibitory activity of natural and synthetic isothiocyanates against representative human microbial pathogens. J. Appl. Microbiol. 115:943-954.

Kurt, S., Güneş, U., Soylu, E.M. 2011. *In vitro* and *in vivo* ungal activity of synthetic pure isothiocyanates against *Sclerotinia sclerotiorum*. Pest Manag Sci. 67(7):869-75.

Liu, T.T. & Yang, T.S. 2010. Stability and antimicrobial activity of allyl isothiocyanate during long-term storage in an oil-in-water emulsion. J. Food Sci. 75(5):C445–C451.

Mastelic, J., Blaževic, I., Kosalec, I. Chemical composition and antimicrobial activity of volatiles from *Degenia velebitica*, a European stenoendemic plant of the Brassicaceae family. 2010. Chem. Biodivers. 7:2755-2765.

Nowicki, D., Maciąg-Dorszyńska, M., Kobiela, W., Herman-Antosiewicz, A., Węgrzyn, A., Szalewska-Pałasz, A., Węgrzyn, G. 2014. Phenethyl isothiocyanate inhibits shiga toxin production in enterohemorrhagic *Escherichia coli* by stringent response induction. Antimicrob Agents Chemother. 58(4):2304-2315.

Pang, Y.H., Sheen, S., Zhou, S., Liu, L., Yam, K.L. 2013. Antimicrobial effects of allyl isothiocyanate and modified atmosphere on *Pseduomonas aeruginosa* in fresh catfish fillet under abuse temperatures. J Food Sci. 78(4):M555-9.

Park, C.M., Taormina, P.J., Beuchat, L.R. 2000. Efficacy of allyl isothiocyanate in killing enterohemorrhagic *Escherichia coli* O157:H7 on alfalfa seeds. Int J Food Microbiol 56(1):13–20.

Radonic, A., Blaževic, I., Mastelic, J., Zekic, M., Skocibušic, M., Maravic, A. 2011. Phytochemical analysis and antimicrobial activity of *Cardaria draba* (L.) desv. volatiles. Chem. Biodivers. 8:1170-1181.

Rhee, M.S., Lee, S.Y., Dougherty, R.H., Kang, D.H. 2003. Antimicrobial effects of mustard flour and acetic acid against *Escherichia coli* O157:H7, *Listeria monocytogenes*, and *Salmonella enterica* serovar Typhimurium. Appl Environ Microbiol 69(5):2959-2963.

Saavedra, M., Borges, A., Dias, C., Aires, A., Bennett, R., Rosa, E., Simões, M. 2012. Antimicrobial activity of phenolics and glucosinolate hydrolysis products and their synergy with streptomycin against pathogenic bacteria. Med. Chem. 6:174-183.

Shofran, B. G., Purrington, S. T., Breidt, F., Fleming, H. P. 1998. Antimicrobial properties of sinigrin and its hydrolysis products. J. Food Sci. 63:621-624.

Ward, S. M., P. J. Delaquis, R. A. Holley, and G. Mazza. 1998. Inhibition of spoilage and pathogenic bacteria on agar and precooked roast beef by volatile horseradish distillates. Food Res. Int. 31:19–26.
